# Uremic Toxins and Frailty in Patients with Chronic Kidney Disease: A Molecular Insight

**DOI:** 10.3390/ijms22126270

**Published:** 2021-06-10

**Authors:** Chia-Ter Chao, Shih-Hua Lin

**Affiliations:** 1 Nephrology Division, Department of Internal Medicine, National Taiwan University Hospital BeiHu Branch, Taipei 10845, Taiwan; b88401084@gmail.com; 2 Graduate Institute of Toxicology, National Taiwan University College of Medicine, Taipei 100233, Taiwan; 3 Nephrology Division, Department of Internal Medicine, National Taiwan University Hospital, Taipei 100255, Taiwan; 4 Nephrology Division, Department of Internal Medicine, National Taiwan University College of Medicine, Taipei 100233, Taiwan; 5 Nephrology Division, Department of Internal Medicine, National Defense Medical Center, Taipei 11490, Taiwan

**Keywords:** advanced glycation endproduct, chronic inflammation, chronic kidney disease, cytokines, frail phenotype, frailty, indoxyl sulfate, oxidative stress, *p*-cresyl sulfate, senescence, uremic toxins

## Abstract

The accumulation of uremic toxins (UTs) is a prototypical manifestation of uremic milieu that follows renal function decline (chronic kidney disease, CKD). Frailty as a potential outcome-relevant indicator is also prevalent in CKD. The intertwined relationship between uremic toxins, including small/large solutes (phosphate, asymmetric dimethylarginine) and protein-bound ones like indoxyl sulfate (IS) and *p*-cresyl sulfate (pCS), and frailty pathogenesis has been documented recently. Uremic toxins were shown in vitro and in vivo to induce noxious effects on many organ systems and likely influenced frailty development through their effects on multiple preceding events and companions of frailty, such as sarcopenia/muscle wasting, cognitive impairment/cognitive frailty, osteoporosis/osteodystrophy, vascular calcification, and cardiopulmonary deconditioning. These organ-specific effects may be mediated through different molecular mechanisms or signal pathways such as peroxisome proliferator-activated receptor γ coactivator 1-α (PGC-1α), mitogen-activated protein kinase (MAPK) signaling, aryl hydrocarbon receptor (AhR)/nuclear factor-κB (NF-κB), nuclear factor erythroid 2-related factor 2 (Nrf2), heme oxygenase-1 (HO-1), Runt-related transcription factor 2 (RUNX2), bone morphogenic protein 2 (BMP2), osterix, Notch signaling, autophagy effectors, microRNAs, and reactive oxygen species induction. Anecdotal clinical studies also suggest that frailty may further accelerate renal function decline, thereby augmenting the accumulation of UTs in affected individuals. Judging from these threads of evidence, management strategies aiming for uremic toxin reduction may be a promising approach for frailty amelioration in patients with CKD. Uremic toxin lowering strategies may bear the potential of improving patients’ outcomes and restoring their quality of life, through frailty attenuation. Pathogenic molecule-targeted therapeutics potentially disconnect the association between uremic toxins and frailty, additionally serving as an outcome-modifying approach in the future.

## 1. Uremic Toxins in Chronic Kidney Disease (CKD): An Introduction to Their Sources and Adverse Influences

A cardinal feature of CKD is the accumulation of uremic toxins (UTs). UTs can be categorized into three groups, water-soluble low molecular weight molecules, middle molecules, and size-varying protein-bound ones [1,2]. Members of the first category include reactive carbonyl compounds, purines, nicotinamides, etc., while those of the second category include proteins (ex. fibroblast growth factor-23 (FGF-23), adiponectin, and leptin) and cytokines (ex. interleukins (ILs), tumor necrosis factor-α (TNF-α), and resistin) [2]. Protein-bound UTs mainly refer to advanced glycation endproducts (AGEs), indole and its derivatives (indoxyl sulfate (IS), indole-3-acetic acid), phenol and its derivatives (*p*-cresyl sulfate (pCS), phenols), polyamines, hippurates, etc. Most of the above compounds have been shown to exhibit a plethora of detrimental effects on cellular physiological processes. The spectrum of UTs is expanding consistently with the advancement of technologies including mass spectrometry and liquid/gas chromatography [3].

The sources of UTs come not only from the perturbed metabolism of organ pathologies, but also from fermentation of dietary constituents by gut microbiota, whose composition is frequently altered during CKD, causing dysbiosis [4]. Accumulating UTs impose pleiotropic effects on a variety of organ systems and affect the metabolism of medications through upregulating hepatic P-glycoprotein expressions and increasing its activity [5]. The UT-related adverse pharmacokinetic effect differs depending on toxin types [6]. The levels of UT closely parallel the risk of adverse outcomes mediated through neurologic, vascular, cardiopulmonary, and musculoskeletal degenerations [7]. In this review, we explain a plausible connection between UTs and frailty and provide the mechanistic and molecular insight for the observed relationship in-between.

## 2. Uremic Toxins and Their Associations with Cellular Degeneration: A Potential Origin of Frailty

The underlying machineries responsible for incident frailty can be complex. Several factors have been implicated during the process of unsuccessful aging, such as anemia resulting from ineffective erythropoiesis [8], architectural bony deterioration [9], vitamin D depletion, etc. Among these factors, many occur in patients with CKD as well. This phenomenon underlies the possibility that shared pathophysiology exists between CKD and frailty, a physical presentation of biological ageing.

### 2.1. Frailty: What Is It and What Causes It?

Frailty is a term used to describe an individual’s vulnerability to environmental and/or endogenous stressors. Advances in assessing approaches have elucidated the epidemiology of frailty and help clarify frailty’s pathophysiological nature and its therapeutic approaches [10]. The most commonly adopted frailty-assessing approaches are the physical frail phenotype and the frail index [11]. Being frail places an individual at an increased risk of adverse outcomes and endows him/her with a tendency to develop incident disability [12]. Moreover, frailty is associated with a rising incidence of adverse events among younger patients with different chronic illnesses, including CKD and end-stage renal disease (ESRD) [13,14].

A plethora of processes have been implicated as the initiators and perpetuators of the frailty. These changes manifest pathologically as sarcopenia, osteoporosis, soft tissue wasting, atherosclerosis/vascular calcification, etc., all of which exhibit a significantly higher incidence in older adults alongside frailty [15,16]. Alterations in sensory functions such as gustatory perception also increase frailty risk [17].

To delve in deeper, molecular signatures related to accelerated aging and incident frailty have been described and proposed before, especially those associated with CKD [18]. We divide cardinal pathological features associated with frailty according to the following categories.

#### 2.1.1. Senescence

Cellular senescence describes the status of irreversible cell cycle stopping precluding tissue regeneration and repair. Senescent cells frequently have a flattened morphology and enlarged shape, associated with an upregulation of p16^INK4A+^, p21^WAF/CIP1^, and p53 [19]. Cells that enter senescence may exhibit phenotypes such as senescence-associated secretory phenotypes (SASPs) and senescent cell anti-apoptotic pathways (SCAPs), serving as both complications and drivers of CKD [20]. Experimentally, vascular tissues are the most well-established sites within which UT-related senescence occurs. UTs were first shown to induce senescence in aortic tissues [21], mediated by the rising oxidative stress, lipid peroxidation, and DNA damages in vascular smooth muscle cells [22]. In endothelial cells, IS altered energy kinetics and suppresses *SIRT1* expression through aryl hydrocarbon receptor (AhR) stimulation, leading to their senescence [23]. The pathogenesis of early vascular aging in patients with CKD also culminates in the adoption of senescent phenotype by vascular tissues [24]. Besides vascular influences, UTs also predispose stem cells to developing senescence. A prior study revealed that pCS stimulated the expressions of *p21* while downregulated cell cycle proteins such as Cdk2, Cdk4, and cyclin D1 in mesenchymal stem cells (MSCs) [25]. Large molecule UTs such as cytokines impair antioxidative defense machineries such as downregulating superoxide dismutase 2 (Sod2) in individuals with CKD, damaging muscular tissues and compromising exercise capacity [26,27]. The reduction of UTs and their downstream effectors have been shown to ameliorate cellular senescence [28], serving as an indirect evidence for the senescence-inducing effect of UTs. UTs also predispose tissues to a senescent phenotype through altering metabolism, nutrient ingestion, and gut dysbiosis. Protein-bound UTs and amines come from various food sources after generation or modulation by gut microbiota, leading to altering DNA methylation status akin to those present during chronological ageing [29].

#### 2.1.2. Mitochondrial Dysfunction

Decline in mitochondrial integrity and its functional compromise constitutes an important explanation for tissue wearing during chronological ageing and disease-induced accelerated aging. Muscular tissue is particularly afflicted; deteriorated muscular proteostasis, reduced ATP production, and abnormal myokine profiles occurs side-by-side with skeletal muscle aging [30]. Mitochondria tend to be fragmented or abnormally enlarged in aging tissues, in combination with lower expressions of mitochondrial fusion proteins (mitofusin (Mfn) and Opa) and fission protein (dynamin related protein (Drp)). Mitophagy, a cellular mechanism aiming to stabilize mitochondrial quality, has also been deranged during the course of frailty development [31]. UTs play an under-recognized role in affecting mitochondrial health, and existing literature frequently addresses the adverse influences exerted by protein-bound ones, such as IS and pCS, such as changes in the extent of autophagy [32].

#### 2.1.3. Stem Cell Exhaustion and Telomere Attrition

MSCs obtained from animals with CKD also present an exhausted phenotype, with a compromised therapeutic efficacy [33]. Uremic milieu induces endoplasmic stress and increases oxidative stress in MSCs of patients with CKD, presenting as a reduced viability, less proliferative capacity, and interruptions in respiratory chain energetics [34]. Stem cell exhaustion may be a vital feature of chronological aging, and uremia appears to confer similar effects on stem cells. Nonetheless, multiple reports have shown that this phenotype of stem cell exhaustion, when accompanied by an accelerated senescence, and alterations in secretomes, can be ameliorated or even reversed by pharmacologic managements [34,35], rendering this phenomenon different from those associated with chronological aging.

Telomeres, the repetitive DNA sequences situated at the end of chromosomes, are another core signature of cellular age and protect host cells from replicative wearing. The attrition of telomeres, or a shortening telomere length, occurs commonly in patients with degenerative disorders including diabetes mellitus and CKD. ESRD patients under dialysis present a telomere-shortening phenotype and a chronically elevated cytokine profile [36]. A progressively lower telomere length has been shown to correlate with an incrementally higher risk of mortality, especially those of cardiovascular origin, in patients with CKD [37]. Telomerase activity has been shown to be reduced in the mononuclear cells of patients under hemodialysis and has become even lower with increasing dialysis vintage [38]. According to a combinatorial analysis of multiple microarray datasets, mononuclear cells from patients with ESRD exhibited downregulations of telomere stabilizer *SERPINE1*/plasminogen activator inhibitor (*PAI*)-1 [39], hinting that UTs participate in altering telomere homeostasis in these patients.

#### 2.1.4. Oxidative Stress and Inflammation

Microscopically, CKD is invariably associated with chronic inflammation and higher oxidative stress. Inflammatory mediators are touted to impair body homeostasis through inducing premature cellular senescence [40]. The phenomenon of “inflammaging”, or the systemic activation of a low grade innate immune-triggered inflammatory response accompanying aging, is similarly observed during uremia [41]. The concurrent presence of reactive oxygen species (ROS) further upregulates the expressions of nuclear factor-κB (*NF-κB*), constituting a vicious cycle of self-perpetuating inflammation [42]. Chronic systemic inflammation, accompanied by an excessive ROS production, underlies the physical degenerative phenotypes observable in CKD patients [24]. These biologic changes, at their advanced stage, may lead to the development of frailty.

Here is a summary of different types of UTs and their influences on the cited categories of frailty pathologic features in Table 1.

### 2.2. Cellular Transport of Uremic Toxins Varies between Cell Types

Susceptibility of each tissue to the adverse influences posed by UTs may differ depending on the types of UTs, tissue perfusion and interstitial fluid levels of UTs, and more importantly, the permeability of cells to UTs. For example, endothelial cell function may be impaired by AGEs binding to surface receptors of AGEs (RAGEs), leading to attenuated survival, migratory ability, and differentiation [43]. Other cells, especially renal tubular epithelial cells, may uptake protein-bound UTs (IS, pCS, hippurates, etc.) through organic acid transporters [44]. The accumulation of intracellular UTs, or the alterations in UT toxicokinetics, may be associated with variable degrees of negative effects outlined above.

## 3. CKD, UTs, and Frailty Are Situated within a Vicious Circle

**Figure 1:**
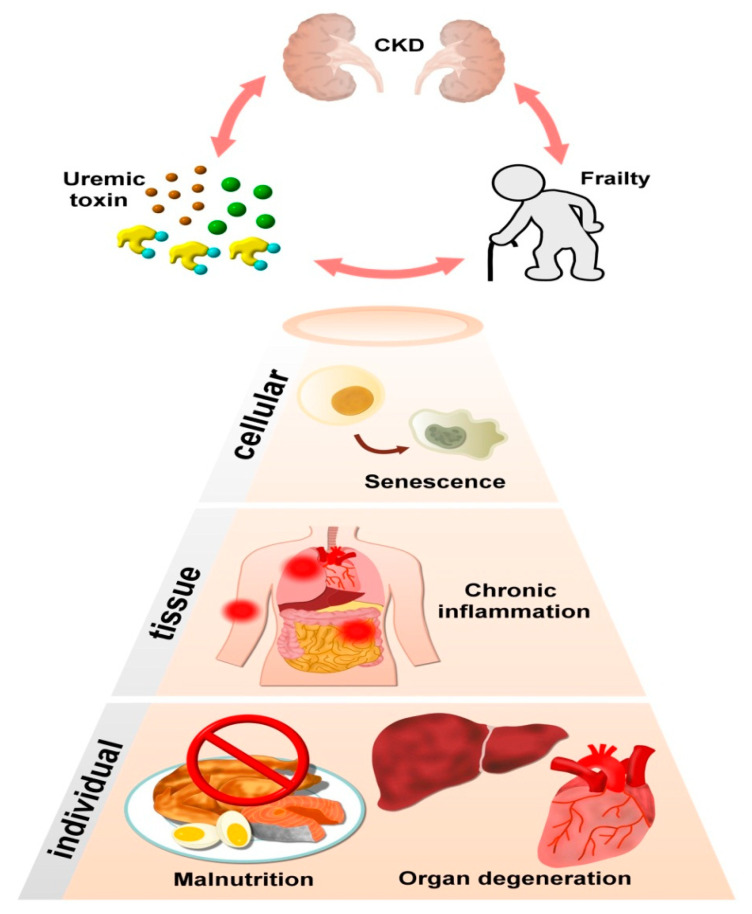
Illustration of potential underlying mechanisms shared between CKD, frailty, and accumulating uremic toxins. CKD, chronic kidney disease.

It is widely acknowledged that patients with CKD have accelerated and premature biological aging [40]. Patients with CKD have an increased incidence of vascular diseases, soft tissue wasting and bone loss, all of which are similarly prevalent in those with chronological aging. Frailty is no exception. A systematic review disclosed that regardless of frailty definitions and the origins of CKD, the prevalence of frailty soars with decreasing estimated glomerular filtration rate (eGFR), being 7% in earlier stage CKD while 73% in those with ESRD [45]. We have shown that 2%–18% of non-dialysis CKD patients had frailty, while more than 50% had prefrailty [13,14,46], supporting the universality of this frailty–CKD relationship. Findings from clinical to experimental reports dictate that UTs likely stand at the pathological intersection between CKD and frailty (Figure 1). Induced cellular senescence is simply one of the variegated dimensions of UT effects on frailty pathogenesis; other features including malnutrition and other organ degenerations, etc. may also be responsible [47]. Furthermore, UTs may exert other organ-/process-specific influences that directly or indirectly set the stage for frailty to occur in patients with CKD.

## 4. Uremic Toxins and Frailty: From Specific Molecular Linkage to Tissue Relevance and Clinical Evidence

**Figure 2:**
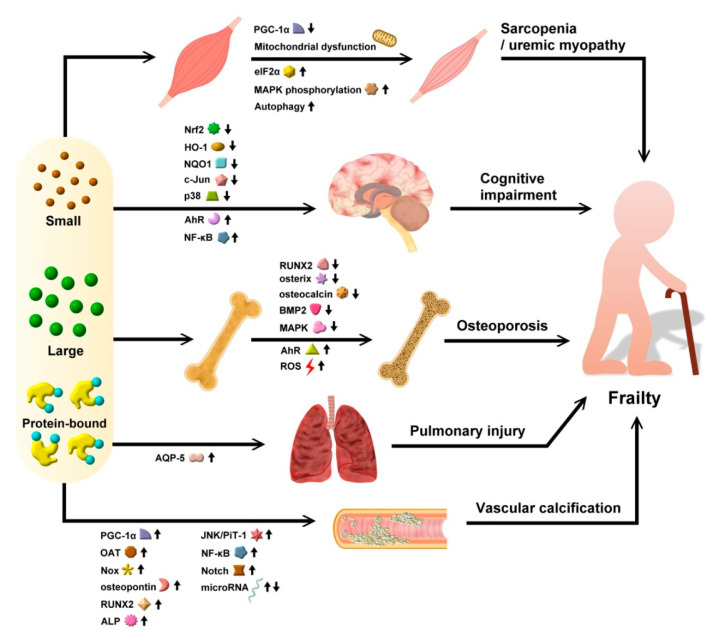
A summary diagram illustrating the putative organ-wide pathogenic relationship between UTs and frailty in patients with CKD. Upward arrows indicate up-regulation, while downward arrows indicate down-regulation. AhR, aryl hydrocarbon receptor; ALP, alkaline phosphatase; AQP, aquaporin; BMP2, bone morphogenic protein 2; CKD, chronic kidney disease; eIF2α, eukaryotic initiation factor 2α; HO-1, heme oxygenase-1; JNK, c-Jun N-terminal kinase; MAPK, mitogen activated protein kinase; NF-κB, nuclear factor-κB; Nox, NADPH oxidase; NQO1, NADPH dehydrogenase quinone 1; Nrf2, nuclear factor erythroid 2-related factor 2; OAT, organic acid transporter; PGC-1α, peroxisome proliferator-activated receptor γ coactivator 1-α; PiT-1, phosphate inorganic transporter 1; ROS, reactive oxygen species; RUNX2, Runt-related transcription factor 2.

Besides the cellular pathophysiology related to UTs-triggered frailty described above, a tissue-oriented perspective may better characterize the pervasive influences introduced by UTs. Indeed, UTs influence the probability of frailty development through modifying multiple preceding events and companions of frailty [48], including sarcopenia, cognitive impairment/cognitive frailty, osteoporosis/osteodystrophy, and cardiopulmonary deconditioning not captured by cellular changes described in Section 3 (Figure 2). Furthermore, anecdotal evidence suggested that full-blown frailty may aggravate renal outcomes, thereby augmenting the accumulation of UTs [14]. These phenomena are addressed in more details below. We therefore provided a summary of molecular changes associated with UTs that may potentially be treatable targets amid the pathogenesis of frailty.

### 4.1. Evidence for Uremic Toxins in Precipitating Sarcopenia

Sarcopenia, either in the form of muscle quantity or quality decrement, are highly prevalent in CKD, with as high as 50% ESRD patients having sarcopenia [49]. Protein-energy wasting, a common scenario in CKD patients, is an important risk factor for soft tissue wasting including muscle loss [50]. The microstructural explanation for CKD-related sarcopenia is uremic myopathy, encompassing all the components of skeletal muscle abnormalities identified in uremic status. Among the uremic milieu, UTs account for a substantial proportion of uremic myopathy.

#### 4.1.1. Protein-Bound UTs

First of all, UTs, especially IS, are myoblast-toxic. Increasing concentrations of IS can cause a greater degree of apoptosis in skeletal muscle myoblasts after 2–3 days of exposure [51]. A group from Japan further showed that myoblasts treated with IS exhibited a down-regulation of peroxisome proliferator-activated receptor gamma coactivator 1-α (PGC-1α) and mitochondrial dysfunction with membrane potential decrease, leading to autophagy and muscle loss [52]. In addition to myoblast survival interference, UTs also dampen their functional capacity. Myoblasts exposed to IS had premature differentiation termination and less myotube formation, with markers such as myoD, myoG, and myosin heavy chain suppressed while *eIF2α* phosphorylation was enhanced [53]. Even if the myotubes are successfully formed, IS-treated myoblasts formed defective ones with a shorter diameter, likely due to an increased mitogen-activated protein kinase (MAPK) phosphorylations and upregulation of atrogin-1 [54]. Anatomically, IS and pCS have been shown to be accumulated in skeletal muscle tissues with a 6- to 10-fold concentration difference compared to non-uremic counterparts, and the levels of muscular UTs correlate linearly with the severity of atrophy in animals [55].

#### 4.1.2. Other UTs

Another UT, AGE, has also been shown to accumulate in the leg muscle of CKD rats and induces irregular muscular contour, fiber size variations, and aberrant capillary rarefactions [56]. Elevated serum AGE levels are shown to parallel the severity of sarcopenia and importantly, frailty, in ESRD patients [56]. Clinical data similarly revealed that among ESRD patients, only asymmetric dimethylarginine (ADMA) but not β2-microglobulin or larger molecular cytokine levels, were associated with poorer muscular performance [57]. Alterations in serum UT levels such as hippuric acid and oxoproline are also close proxies of physical inactivity in those of advanced age [58]. Despite the rare findings of a lack of associations between UTs and sarcopenia in small scale studies [59], overwhelming clinical reports derive consistent results that UT levels closely follow the severity of skeletal muscle loss, increasing the susceptibility to developing frailty, in CKD/ESRD patients [56,57,58].

### 4.2. Evidence for Uremic Toxins in Predisposing Patients to Cognitive Impairment/Frailty

An emerging subcategory of frailty, termed cognitive frailty, has been defined recently. Conceptually, cognitive frailty intends to describe the reversible state of a reduced cognitive reserve in the absence of dementia or other brain disorders [60]. Cognitive frailty frequently accompanies physical frailty. A recent meta-analysis revealed that patients with cognitive frailty was associated with 93% and more than 2-fold higher risk of mortality and dementia, respectively, compared to those without among older adults [61]. Patients with CKD are also at risk of developing encephalopathy and cognitive impairment. It is expectable that UTs play an indispensable role in this neuropathological process associated with renal insufficiency.

#### 4.2.1. Protein-Bound UTs

In vitro studies found that IS-treated astrocytes and glial cells had downregulation of nuclear factor erythroid-derived 2-like 2 (*Nrf2*), heme oxygenase-1 (*HO-1*), and nicotinamide adenine dinucleotide phosphate (NADPH) dehydrogenase quinone 1 (*NQO1*), but upregulation of *AhR* and *NF-κB* [62]. IS is directly neurotoxic, with a dose-dependent neuronal death upon exposure, and at the same time creates an unfriendly environment in the central nervous system [63]. In addition, the *MAPK* pathway, *c-Jun* signaling, and *p38* were also suppressed in astrocytes subjected to IS exposure, accompanied by mitochondrial membrane disruption [63]. In the CKD animal, the concentrations of IS increased universally across all parts of brain except hypothalamus, and a gradient elevation in brainstem levels could be observed following higher IS dosages [64]. In addition, chronically increased IS reduced neurotransmitters such as norepinephrine, serotonin, and dopamine in brainstem, increasing stress sensitivity and lowering locomotor activities, both of which were core characteristics of cognitive frailty [64]. There are also reports identifying IS in cerebrospinal fluids [65]. Nephrectomized mice receiving pCS for 7 weeks exhibit aberrant behavior suggesting cognitive impairment and neuropsychiatric disturbances [66]. IS entered central nervous system through the abnormal blood brain barrier with an increased permeability and induced neurobehavioral alterations through binding to the overexpressed AhR in cerebral tissues among CKD rats [67]. Exposure to IS also results in the suppression of neural stem cell activity and a lower level of brain-derived neurotrophic factors (BDNFs), amenable to correction by AhR antagonism [65].

In clinical studies, higher serum IS concentrations were associated with poorer neuropsychologic performance among patients with stage 3 CKD [68]. Higher serum indole-3 acetic acid also raises the risk of cognitive impairment among patients with ESRD [69]. We previously showed that frailty in patients with CKD was associated with a higher risk of delirium/cognitive impairment as well [70]. From the above in vitro, in vivo, and clinical findings, UTs pose a direct neurotoxic effect and are associated with adverse neurobehavioral features in patients with CKD.

#### 4.2.2. Other UTs

Despite the buffering function of blood–brain barrier and the low cerebrospinal fluid-to-plasma ratio of UTs, the accumulation of non-protein bound UTs in cerebral tissues follows that in plasma [71]. Uremic solutes, such as methylglyoxal, have been shown to compromise neuronal cell viability and increase oxidative stress [72]. Besides direct neurotoxic effects, non-protein bound UTs such as uric acid, lanthionine, or methylguanide may affect blood pressure-regulating neurons, increase vascular ROS and alter vessel tone, predisposing individuals to hypertension and developing cerebrovascular diseases [73]. Homocysteine, a water-soluble UT, and metabolites from the kynurenine pathway were shown to exert a prothrombotic effect on vasculature, disrupt hemostasis, and potentially contribute to the risk of stroke [74]. Clinically, increased serum levels of homocysteine are associated with a higher risk of cognitive impairment among ESRD patients [75]. A majority of inflammatory mediators, including interleukins, induces cerebral inflammation and oxidative damages, causing excitotoxic injuries and the resultant neurodegeneration [76]. An enhanced clearance of middle molecules also correlates closely with a better cognitive performance and executive function in ESRD patients [77].

### 4.3. Evidence for Uremic Toxins in Inducing Osteoporosis

Osteoporosis, or reduced bone mineral density or qualitative disturbance, is an important complication related to uremia. For those with earlier stage of CKD (e.g., 1–3), the pathology of bone may be similar to that of the general population, while for those with advanced CKD (e.g., 4–5D), the condition becomes complicated with difficult-to-predict osteo-pathology accompanying biochemical abnormalities [78]. These changes arise from multiple endocrinologic and microenvironmental changes due to renal function decline [79].

#### 4.3.1. Protein-Bound UTs

IS and pCS have been shown to compromise MSC osteoblastic differentiation and osteoblastogenesis, presenting as decreased expressions of *RUNX2*, through activating AhR signaling and suppressing MAPK pathways [80,81]. In addition, IS can downregulate osterix, osteocalcin, and bone morphogenetic protein 2 (*BMP2*), thereby reducing bone formation while simultaneously reduce osteoclast formation through lowering receptor activator of NF-κB ligand (*RANKL*) expressions [82]. IS is toxic to osteoblasts in vitro, through uptake by organic anion transport (OAT) and ROS induction [83]. These mechanisms are expected to result in a low bone turnover status, typical of the adynamic bone disease in patients with advanced CKD. Several reports already indicate that UT levels correlate with bone histomorphology in CKD patients, and osteoporosis significantly increases the risk of developing frailty. From a small cohort of predialysis CKD patients, a positive relationship between serum IS levels and bone fibrosis volume was clearly found [84]. We also showed that among a large CKD population, the presence of osteoporosis introduced 20% higher risk of frailty over 3.5 years of follow-up [9], affirming the tight association between osteoporosis and frailty in CKD patients. 

#### 4.3.2. Other UTs

Important UT species participating in the pathogenesis of uremic osteoporosis include parathyroid hormone, FGF-23, and others. Treating MSCs with uremic milieu and selected UTs, such as parathyroid hormone, asymmetric dimethylarginine, and homocysteine, attenuates the tendency of MSCs toward osteogenesis [85]. A UT, phenylacetic acid, was shown to impair bony response to parathyroid hormone and increase the possibility of adynamic bone disorder [86].

### 4.4. Evidence for Uremic Toxins in Causing Cardiopulmonary Deconditioning

#### 4.4.1. Cardiovascular System

UTs are perceived as instrumental drivers of cardiovascular events in CKD patients. Traditional cardiovascular risk features have a higher incidence and prevalence in these patients, while atypical risk factors compound the risk further. Among atypical factors, UTs are the most important ones.

##### Protein-Bound UTs

Experimentally, cardiomyocytes exposed to pCS exhibited a decreased frequency of spontaneous contraction and irregularity in beating through pCS-induced rise in intracellular calcium levels, activation of protein kinase Cα (PKCα), and disassembly of gap junction protein Cx43 [87]. At high concentrations, IS is toxic to cardiomyocytes, vascular smooth muscle cells (VSMCs), and endothelial cells (ECs) [88]. In CKD mice, more than 10-fold higher IS levels could be detectable within their cardiac tissues, significantly higher than those within other tissues [55]. Compared to the heart, the influences of UTs on vascular tissues are more diverse and prominent. VSMCs treated with IS have been shown repeatedly to exhibit a procalcific tendency with trans-differentiation toward an osteoblast-like phenotype [89]. This osteoblast-like change, manifested clinically as vascular calcification, involves the upregulation of *OAT*, NADPH oxidase (*Nox*), osteopontin, *RUNX2*, and alkaline phosphatase. Potential mediators and countering responses of this calcific tendency include prelamin A [22], JNK/PiT-1, SET7/9 [90], PI_3_K/Akt/NF-κB [91], Notch signaling [92], and epigenetic regulators (ex. miR-155, miR-125b, and more) [93,94]. Using models of in vitro VSMC culture and calcified aortas from CKD rats, we demonstrated that miR-125b and miR-378a-3p might participate in the pathogenesis of IS-induced vascular calcification [95,96]. Occurrence of uremic vascular calcification is associated with vascular stiffening, increased cardiac afterload, and a higher risk of heart failure [97]. This adverse vascular remodeling, if involved in aorta, cerebral vessels, and limb arteries, can culminate in incident stroke and peripheral vascular disease, compromising physical activities and finally causing frailty. The relationship between UTs, VC, and left ventricular remodeling has been validated in prior clinical reports [98]. We have similarly demonstrated that VC portended a higher risk of frailty [16].

##### Other UTs

Small molecule UTs including inorganic phosphate and even calcium are well known precipitators of VC, both clinically and experimentally [97,99]. Phosphate treatment involving VSMCs induces an upregulation of *PiT-1*, *PiT-2*, *NF-κB*, and Wnt/β-catenin signaling, leading to a higher expression of *RUNX2*, a core determinant of osteoblastic differentiation process [100]. RAGEs also play an important role in VC pathogenesis; *RAGE* knock-out mice with CKD had significantly less VC severity through a downregulation of *PiT-1* [101]. Large molecular UTs including TNF-α promotes VSMC biomineralization through upregulating *ERK*/*AP1*/*c-Fos* expressions and microRNA dysregulation, thereby aggravating VC [97,102]. FGF-23, a hormonal UT, has been shown to increase the risk of cardiovascular events and precipitate VC, through altering the expression of phospholipase C (*PLC*)/calcineurin/nuclear factor activating factor (*NFAT*) pathway and vitamin D signaling [103].

#### 4.4.2. Pulmonary System

Lung injury is often regarded as a secondary event resulting from acute kidney injury. Although direct evidence of UTs on pulmonary tissues or constituent cells is relatively limited, a preliminary study showed that rats with AKI developed pulmonary interstitial thickening and increased pulmonary aquaporin-5 (*AQP-5*) expressions accompanied by elevated serum IS levels [104]. The reduction of IS using oral adsorbents in these rats could attenuate pulmonary *AQP-5* expressions and ameliorate pulmonary pathology, serving as an indirect evidence for UT-induced pulmonary abnormalities. Further studies are still needed to confirm that UTs can induce frailty through direct pulmonary injuries and respiratory insufficiency.

A brief summary of the above reports is provided in Table 2.

### 4.5. Frailty Accelerates Renal Progression and Possibly Increased UT Levels

Frailty and UTs can be mutually inductive, constituting a vicious cycle (Figure 2). The retention of several categories of UTs, such as inflammatory cytokines, can alter appetite-regulating hormones (leptin and ghrelin), thereby affecting oral intake in CKD patients [105]. Decreased oral intake and malnutrition are important contributors to frailty in this population [48]. On the other hand, once frailty occurs, its sequel such as oral frailty further results in less dietary intake, malnutrition potentially volume depletion, and declining renal functions [7] followed by the accumulation of UTs. Using a population-based cohort of CKD patients, we discovered that patients with frailty were associated with a 20% higher risk of developing ESRD within 4 years than those without, independent of confounding factors such as demographic features, comorbidities, and relevant medications [14]. We believe that this pathobiological connection should be monitored and prevented if frailty occurs in CKD patients.

## 5. Mediators of Organ Degeneration and Frailty as Emerging Diagnostics and Therapeutic Targets

As suggested above, multiple cell type-specific signaling pathways may participate in the pathogenesis of frailty. It would be tempting to presume that these molecules may serve as markers of frailty, and that pharmaceuticals or natural compounds exhibiting activities for upholding or suppressing these pathways can be beneficial for lowering frailty risk in vulnerable individuals. Results from several studies may provide us with encouraging hints. In a prior attempt, we showed that in older adults, circulating miR-125b predicted the risk of VC, an important risk factor for frailty, in community-dwelling older adults [106,107]. A recent study showed that 2 weeks of metformin, a potential MAPK inhibitor, administration could improve myoblast functions in older adults through histone and chromatin remodeling [108]. Astaxanthin, an emerging senolytic, could attenuate vasculopathy through upregulating antioxidant enzyme SOD2 [99]. These findings support the notion that molecular mediators of multiple senescence-associated phenotypes may have potential clinical utility.

## 6. Future Perspectives

The existing literature has provided us with a robust source of data supporting the relationship between uremic toxins and frailty pathogenesis, ranging from epidemiological linkage to experimental evidence testing the direct contribution of UTs to multiple frailty predecessors. Since the presence of frailty greatly increases the risk of adverse outcomes and rising healthcare costs related to hospitalization/emergency visits [109,110], multiple strategies are devised to lower the probability of frailty occurrence and to attenuate its severity. Exercise, nutritional interventions, multimodality approach, and the comprehensive geriatric assessment/care have been advocated as potential treatments for frailty, while pharmacologic management still needs more evidence to support [111].

It is tempting to presume that UT reduction may benefit CKD patients in terms of frailty amelioration. Oral adsorbents or dedicated dialysis modalities for UT reduction may be practical approaches [2,112]. A broader concept of gastrointestinal decontamination, such as inhibiting NaPi, is readily available for use. Dietary modification such as very low protein diet with or without supplemental nutrients may also hold promise in averting renal function decline and lowering UT levels [113]. In addition, a more intensive reduction of uremic toxins, such as frequent hemodialysis or a prolonged dialysis regimen may offer incremental benefit for UT lowering and putatively for frailty amelioration. Data from the frequent hemodialysis network indicated that such approach could further lower multiple types of UTs by 15%, although protein-bound ones exhibited minimal changes [114]. Alternatively, the use of newer designs of artificial kidneys may be a promising strategy to lower middle molecule UTs, without altering nutritional status in patients with ESRD [115]. A more comprehensive understanding of this UT-frailty association may assist in uncovering novel strategies to ameliorate frailty in CKD population.

## Figures and Tables

**Table 1:** Classification and potential adverse effects of uremic toxins.

Category	Subtype	Species	Adverse Effects				
			Senescence	Inflammation	OS	Mt Dysfunction	SC Damages
Free, water soluble, LMW molecules	Reactive carbonyl group	2-Hexenal		(+)	(+)	(+)	
		Hexanal		(+)	(+)	(+)	
		2-Nonenal	(+)	(+)	(+)	(+)	
		Nonanal		(+)	(+)		
		2-Octenal	(+)		(+)		(+)
		4-OH-Hexenal		(+)	(+)		
		4-OH-Nonenal		(+)	(+)	(+)	
		Malondialdehyde	(+)	(+)	(+)		
	Nicotinamide	4-Pyridone-3-carboxamide-1β-ribonucleoside-triphosphate (4PYTP)		(+)	(+)		
		4-Pyridone-3-carboxamide-1β-ribonucleoside-monophosphate (4PYMP)		(+)	(+)		
	Purine	8-hydroxy-2’-deoxyguanosine	(+)	(+)	(+)	(+)	(+)
		Hypoxanthine		(+)	(+)	(+)	
		Neopterin	(+)	(+)	(+)		(+)
		Uric acid	(+)	(+)	(+)	(+)	(+)
	Guanidine	α-Keto-δ-guanidinovaleric acid		(+)			
		Guanidinoacetic acid		(+)	(+)	(+)	
		Guanidinosuccinic acid		(+)	(+)		
		Methylguanidine	(+)	(+)	(+)	(+)	
	Amine	ADMA	(+)	(+)	(+)	(+)	(+)
		Dimethylamine		(+)	(+)	(+)	
		Monomethylamine		(+)	(+)	(+)	
Middle molecules	Protein	α1-acid glycoprotein		(+)			
		α1-microglobulin		(+)	(+)		
		β2-microglobulin	(+)	(+)	(+)		
		Adiponectin	(+)	(+)	(+)	(+)	
		Complement factor D	(+)	(+)	(+)		
		FGF-23	(+)	(+)	(+)		(+)
		Leptin	(+)	(+)	(+)	(+)	
		Parathyroid hormone		(+)	(+)	(+)	
		Retinol binding protein		(+)	(+)	(+)	
		Soluble intracellular adhesion molecule-1	(+	(+)	(+)		
	Cytokine	Interleukin-6	(+)	(+)	(+)	(+)	(+)
		Interleukin-8	(+)	(+)	(+)	(+)	(+)
		Resistin	(+)	(+)	(+)	(+)	(+)
		Tumor necrosis factor-α	(+)	(+)	(+)	(+)	(+)
Protein-bound molecules	Reactive carbonyl group	Acrolein	(+)	(+)	(+)	(+)	(+)
	AGE	Carboxymethyllysine	(+)	(+)	(+)	(+)	(+)
		Pentosidine	(+)	(+)	(+)		
	Hippurate	Hippuric acid		(+)	(+)		
	Amino acid	Homocysteine	(+)	(+)	(+)	(+)	(+)
	Indole	Indoxyl sulfate	(+)	(+)	(+)	(+)	(+)
		Indole-3-acetic acid	(+)	(+)	(+)	(+)	
		Kynurenic acid		(+)	(+)	(+)	(+)
	Phenol	*p*-Cresylsulfate	(+)	(+)	(+)	(+)	(+)
	Polyamine	Spermidine		(+)	(+)		

ADMA, asymmetric dimethylamine; AGE, advanced glycation endproduct; FGF-23, fibroblast growth factor-23; LMW, low molecular weight; Mt, mitochondria; OS, oxidative stress; SC, stem cell.

**Table 2:** Experimental findings connecting uremic toxins to frailty pathogenesis based on different cell types.

Toxin Types	UT Species	Cell Type Involved	Molecular Mediators
Protein-bound	Indoxyl sulfate	Muscle/myoblasts	PGC-1*α* downregulation
		Muscle/myoblasts	myoD, myoG, MHC downregulation
		Muscle/myoblasts	MAPK phosphorylation increase, atrogin-1 upregulation
		Brain/astrocytes, glial cells	Nrf2, HO-1, NADPH dehydrogenase quinone 1 down-regulation
		Brain/astrocytes, glial cells	AhR, NF-κB upregulation
		Brain/astrocytes	MAPK, c-Jun, p38 downregulation
		Brain/neural stem cells	BDNF down regulation
		Bone/MSCs	MAPK downregulation but AhR activation
		Bone/osteoblasts	Osterix, osteocalcin, BMP2 downregulation
		Bone/osteoclasts	RANKL downregulation
		Vessel/VSMCs	Prelamin A, JNK, PiT-1, SET7/9, PI_3_K/Akt/NF-κB, Notch upregulation
		Lung/pneumocytes	AQP5 upregulation
	*p*-cresol	Bone/osteoblasts	JNK/p38 activation
		Heart/cardiomyocytes	PKC*α* activation, Cx43-related gap junction disintegration
Small molecular	Methylglyoxal	Brain/neurons	ROS activation
	Phenylacetic acid	Bone/osteoblasts	PTH response impairment
	Phosphate	Vessel/VSMCs	PiT-1, PiT-2, NF-κB, Wnt/β-catenin
Large molecular	TNF-*α*	Vessel/VSMCs	ERK/AP1/c-Fos upregulation

AhR, aryl hydrocarbon receptor; AP-1, activator protein-1; AQP5, aquaporin 5; BDNF, brain-derived neutrophic factor; BMP2, bone morphogenic protein 2; ERK, extracellular signal-regulated kinase; HO-1, heme oxygenase-1; JNK, c-Jun N-terminal kinase; MAPK, mitogen activated protein kinase; MHC, major histocompatibility complex; MSC, mesenchymal stem cell; NF-κB, nuclear factor-κB; Nrf2, nuclear factor erythroid 2-related factor 2; OAT, organic acid transporter; PGC-1α, peroxisome proliferator-activated receptor γ coactivator 1-α; PI_3_K, phosphoinositide 3-kinase; PiT, phosphate inorganic transporter; PKCα, protein kinase C α; PTH, parathyroid hormone; RANKL, receptor activator of nuclear factor κB ligand; ROS, reactive oxygen species; RUNX2, Runt-related transcription factor 2; TNF, tumor necrosis factor; VSMC, vascular smooth muscle cell.

## Data Availability

There is no new datum generated in this review.
